# 

**Published:** 1906-03

**Authors:** 


					C OiN TEN TS,
Fage
Arrti -Tuberculous Vaccines and Sera.
By J. M. Fortescue-
Brickdale. M.A., M.D. Oxon.
The Prognosis of Pulmonary Tuberculosis.
By J. J. S. Lucas,
M.D., B.A.Lond.
12
Why Defective Nasal Respiration Impedes Growth and Develop-
ment.
Bv P- Watson Williams, M.D. Lond. ...
17
Some Effects of Fright in Medicine.
By J. R. Charles, M.A.,
M.D.Camb., M.R.C.P.
23
Colitis Polyposa.
By Carey Coombs, M.D., B.S.Lond.
3i
Notes of a Case of Acute Yellow Atrophy of the Liver.
By
F. H. Edgeworth, M.B.Cantab., D.bc. Lond.
37
A Case of Acute Yellow Atrophy of the Liver in a Child.
By
Bertram M. H. Rogers, 13.A., M.D., r>.Ch. Oxon.
4?
Notes on a Case of Splenomegalic Biliary Cirrhosis in a Boy
aged six years.
By Edward Cecil Williams, M.B. Cantab.
42
Clinical Note on a Case of Epithelioma of Tongue in a Young
Woman.
By C. Hamilton whitkford, m K.L>.b.,
45
Progress of the Medical ociences.
Medicine.
R. Shingliton Smith, M.D. Lond., F.R.C.P.
47
Surgery.
J. Lacy Firth, M.D.Lond., F.R.C.S.
52
Dermatology.
W. Kenneth Wills, M.A., M.B., B C. Cantab. ..
57
Reviews of Books
6i
Editorial Notes
7?
Notes on Preparations for the Sick
Si
The Library of the Bristol Medico-Chirurgical Society ...
85
Meetings of Societies
88
Obituary Notices ...
93
Local Medical Notes
95

				

